# Brief research report: WGCNA-driven identification of histone modification genes as potential biomarkers in AQP4-Associated optic neuritis

**DOI:** 10.3389/fgene.2024.1423584

**Published:** 2024-08-22

**Authors:** Yuan Cao, Wen Yao, Fang Chen

**Affiliations:** Department of Ophthalmology, Northern Jiangsu People’s Hospital, Yangzhou University, Yangzhou, China

**Keywords:** neuromyelitis optica spectrum disorder (NMOsd), aquaporin −4 (AQP4), weighted gene co-expression network analysis (WGCNA), biomarker, histone modification

## Abstract

**Introduction:**

Neuromyelitis Optica spectrum disorder (NMOSD) is an autoimmune disease characterized by anti-aquaporin-4 (AQP4) auto-antibodies. The discovery of antibodies AQP4 and myelin oligodendrocyte glycoprotein (MOG) has expanded our understanding of the pathogenesis of neuromyelitis optica. However, the molecular mechanisms underlying the disease, particularly AQP4-associated optic neuritis (AQP4-ON), remain to be fully elucidated.

**Methods:**

In this study, we utilized Weighted Gene Co-expression Network Analysis (WGCNA) to investigate the transcriptomic profiles of peripheral blood samples from patients with AQP4-ON and MOG-positive optic neuritis (MOG-ON), compared to healthy controls.

**Results:**

WGCNA revealed a brown module (ME brown) strongly associated with AQP4-ON, which correlated positively with post-onset visual acuity decline. A total of 132 critical genes were identified, mainly involved in histone modification and microtubule dynamics. Notably, genes HDAC4, HDAC7, KDM6A, and KDM5C demonstrated high AUC values in ROC analysis, indicating their potential as biomarkers for AQP4-ON.

**Conclusion:**

Our findings provide novel insights into the molecular signature of AQP4-ON and highlight the potential of systems biology approaches in identifying biomarkers for NMOSD. The identified histone modification genes warrant further investigation for their role in disease pathogenesis and as therapeutic targets.

## Introduction

Neuromyelitis Optica spectrum disorder (NMOSD) is an autoimmune inflammatory disease of the central nervous system, characterized by optic neuritis and acute transverse myelitis, which can lead to irreversible visual impairment and a range of neurological deficits (Tian et al., 2020; Carnero Contentti and Correale, 2021). The identification of pathogenic antibodies to aquaporin-4 (AQP4) has marked a significant milestone in NMOSD research, elucidating the pivotal role of humoral immunity in disease pathogenesis (Lennon et al., 2005; Prasad and Chen, 2019).

The discovery of myelin oligodendrocyte glycoprotein (MOG) antibodies in a subset of patients with similar autoimmune demyelinating syndromes has expanded our understanding of the disease spectrum (Wynford-Thomas et al., 2019). This discovery has resulted in the recognition of a distinct entity: myelin-oligodendrocyte glycoprotein antibody-associated disease (MOGAD) (Banwell et al., 2023). Despite these insights, the molecular intricacies of NMOSD, particularly the complex interplay between adaptive and innate immune responses, are not fully characterized. The delineation of the mechanistic nuances between NMOSD and MOGAD is imperative for advancing our comprehension of these conditions.

The emergence of high-throughput transcriptomic technologies has catalyzed a paradigm shift in the dissection of NMOSD’s intricate genetic architecture (Liu et al., 2021; Miao et al., 2023). Transcriptome-wide association studies (TWAS) have shed light on numerous candidate genes and pathways involved in immune cell trafficking, cytokine signaling, and neuroinflammation, all of which are implicated in the disease’s etiology (Mai et al., 2023; Schlosser et al., 2023). In this context, bioinformatics strategies, particularly Weighted Gene Co-expression Network Analysis (WGCNA), have proven indispensable in revealing the underlying gene expression patterns associated with disease phenotypes (Langfelder and Horvath, 2008). WGCNA enables the identification of gene modules whose expression is tightly correlated with clinical phenotypes, offering a holistic perspective on disease mechanisms (Zhao et al., 2010).

WGCNA has been effectively utilized to identify gene modules associated with a spectrum of neurological disorders, including multiple sclerosis and Alzheimer’s disease (Wang et al., 2022; Zhao et al., 2023). However, its application in demyelinating diseases, especially in the context of AQP4 and MOG antibody-associated optic neuritis pathogenesis, is yet to be fully explored.

In this study, we harnessed the analytical capabilities of WGCNA to conduct a systematic exploration of the transcriptomic profiles of peripheral blood samples from patients with AQP4-positive optic neuritis (AQP4-ON) and MOG-positive optic neuritis (MOG-ON), juxtaposed with those from healthy controls. By integrating gene module analysis with clinical phenotyping, we endeavored to uncover key genes and pathways that could serve as therapeutic targets or biomarkers for NMOSD. Our study contributes to the molecular characterization of NMOSD and underscores the potential of systems biology in deciphering the complexities of autoimmune diseases affecting the central nervous system.

## Materials and methods

### Data source and preliminary data processing

In the present study, peripheral blood transcriptome data from normal volunteers and patients with optic neuritis were sourced from the Gene Expression Omnibus (GEO) database (www.ncbi.nlm.nih.gov/geo), with the dataset identified by the accession number GSE226808. The dataset includes seven healthy controls, six AQP4-ON patients, and seven MOG-ON patients. Patients were labeled sequentially according to their clinical groups (Healthy control from H1 to H7, AQP4-ON from A1 to A6, and MOG-ON from M1 to M7). Phenotype data of the patients used in the study can be found in the related article and were listed in Supplementary Table S1. The R software (Version 4.3.1) was utilized for data retrieval, followed by standardization of the data to facilitate subsequent analyses.

### WGCNA analysis and key gene selection

Quality control and module division of the standardized sequencing data were performed using the *WGCNA* package (Version 1.72) and *flshClust* package (Version 1.01) within the R software. Modules with a correlation coefficient greater than 0.7 were merged. Subsequently, the correlation between gene modules and clinical phenotypes was analyzed using the R packages *stats* (Version 4.3.1), *ggplot2* (Version 3.4), and *ggpubr* (Version 0.6); intersection analysis of genes was accomplished with the Venn Diagram package (Version 1.7.3). It should be noted the best corrected visual acuity (BCVA) in this study was presented and analyzed in the form of a logarithm of minimal angle resolution (logMAR).

### Enrichment analysis and protein-protein interaction (PPI) network analysis

Gene Ontology (GO) enrichment analysis was conducted and visualized using the R package *clusterProfiler* (Version 4.10.0) for the key genes identified through bioinformatics screening. A Protein-Protein Interaction (PPI) network for the key genes was constructed using the *STRING* database (www.string-db.org) and visualized using *Cytoscape* software (Version 3.10).

### ROC curve analysis

The R package *pROC* (Version 1.18) in conjunction with *ggplot2* was employed to plot the ROC curves for individual genes and to calculate the 95% confidence intervals (95% CI)for the AUC values.

### Statistical analyses

Statistical analyses were performed to compare data across groups. For comparisons between the two groups, we employed Student’s t-test to evaluate the significance of differences. When extending our analysis to multiple groups, Turkey’s multiple comparison methods were used to adjust for the increased risk of Type I errors. All statistical tests were conducted with a significance level set at *P* < 0.05, indicating a threshold for statistical significance, unless otherwise noted. All tests were two-tailed.

## Results

### WGCNA analysis reveals modules associated with AQP4-associated optic neuritis

**FIGURE 1:**
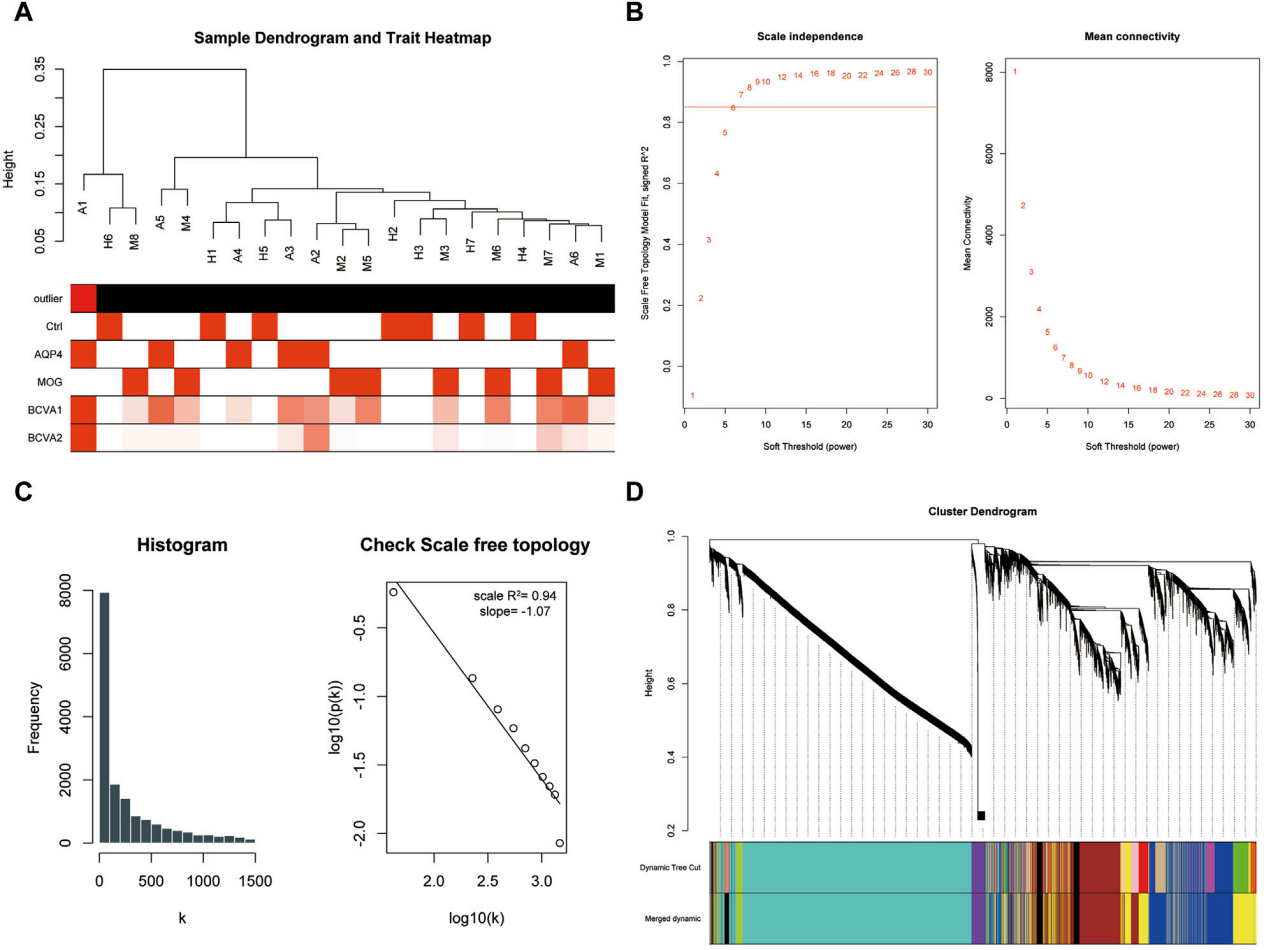
WGCNA analysis constructed a scale-free network and distinguished gene modules. **(A)** Sample dendrogram illustrating the hierarchical clustering of samples in the study. Outliers were identified and excluded from further analysis. **(B)** The scale-free topology fit plot showed the R-squared value approaching 0.9 at a soft-thresholding power of 9, indicating a good fit to a scale-free network model. **(C)** Histogram and correlation analysis further supported the scale-free network distribution. **(D)** The module assignment plot showed seven distinct gene modules resulting from the WGCNA analysis.

The dataset GSE226808, encompassing peripheral blood RNA-sequencing samples from patients with aquaporin-4 positive optic neuritis (AQP4-ON), myelin oligodendrocyte glycoprotein (MOG) antibody positive optic neuritis (MOG-ON), and healthy controls, was utilized in this study. Following the exclusion of lowly expressed genes and standardization of sequencing data through z-score normalization (Supplementary Figure S1), we employed Weighted Gene Co-expression Network Analysis (WGCNA) to identify modules correlated with the pathogenesis of neuromyelitis optica. The construction of a sample dendrogram identified sample A1 as the outlier and excluded sample A1 from further analyses. (Figure 1A), with the remaining samples advancing to subsequent analyses. At a soft-thresholding power of 9, the R-squared value for the entire weighted co-expression network approached 0.9, indicating proximity to a scale-free network distribution (Figure 1B). This was further corroborated by histogram analysis and correlation analysis (Figure 1C). Consequently, a soft threshold of 9 was selected for WGCNA, leading to the consolidation of highly intercorrelated gene modules. Ultimately, all sequenced genes were categorized into seven distinct modules (Figure 1D).

### Functional annotation of key genes and analysis of hub genes in AQP4-associated optic neuritis

**FIGURE 2:**
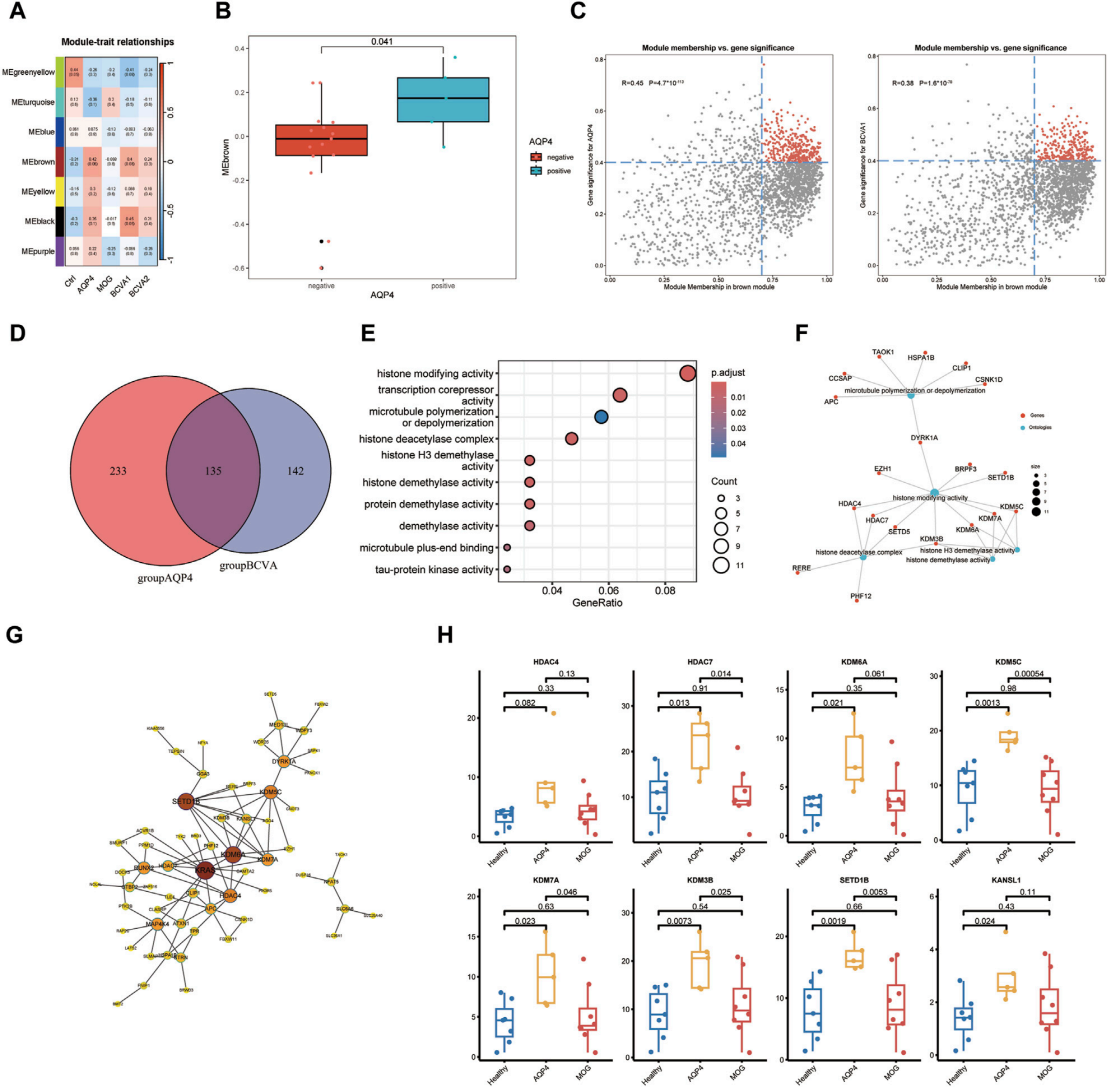
Functional Annotation and Analysis of AQP4 positive Optic Neuritis. **(A)** Heatmap analysis depicting correlation between the seven modules and the clinical phenotypes. **(B)** Boxplot showing significant discrepancy of Module scoring across two sample types (AQP4 negative and AQP4 positive). **(C)** Scatter plots showing the correlation between gene significance (GS) and module membership (MM) for genes within the MEbrown module, with selection criteria of MM > 0.7 and GS > 0.4. The selected genes were marked in red. **(D)** Venn diagram representing the intersection of selected genes identified from the correlation scatter plots, resulting in 135 key genes. **(E)** Functional annotation analysis indicating the predominant biological processes in which the identified key genes are involved. **(F)** Genes associated with histone deacetylase activity (HDAC4, HDAC7, SETD5) and histone demethylase activity (KDM6A, KDM7A, KDM3B, KDM5C) as identified in the study. **(G)** Protein-protein interaction (PPI) network analysis highlighting the central positions of key genes within the network. **(H)** Transcripts per million (TPM) expression levels of the identified key genes in the peripheral blood of AQP4-ON patients compared to MOG-ON patients and healthy controls.

In pursuit of peripheral blood transcriptomic markers significantly associated with AQP4 positive optic neuritis, a phenotypic correlation analysis was conducted on the gene modules identified in the preceding analysis. Post-onset best-corrected visual acuity (BCVA1), best-corrected visual acuity after treatment (BCVA2), and disease groups of the patients (AQP stands for AQP4+ON, MOG stands for MOG + ON and Ctrl for Healthy controls) were included in the correlation analysis. It was observed that the brown module (MEbrown) demonstrated a significant correlation with the AQP4 positive optic neuritis phenotype and a positive correlation with the extent of post-onset visual acuity decline (Figures 2A, B). Subsequently, genes within the MEbrown module were selected for further analysis. A correlation analysis between gene significance (GS) and module membership (MM) for all genes within the MEbrown module was performed to ascertain whether MM values were closely associated with the onset of AQP4-ON and the decline in visual acuity post-onset. The analysis revealed a significant correlation between Module membership values of genes within the brown module and the gene significance related to AQP4 phenotype and visual acuity decline. By setting the MM > 0.7 and GS > 0.4 as the selection criteria, the key genes from the two scatterplots were identified (Figure 2C). Following the intersection of key genes from the aforementioned correlation scatter plots, a total of 132 critical genes were identified in the Venn diagram (Figure 2D). Functional annotation analysis indicated that these genes are predominantly involved in biological processes such as histone modification, microtubule polymerization/depolymerization, and transcriptional corepressor activity (Figure 2E). Notably, genes including HDAC4, HDAC7, and SETD5 were associated with histone deacetylase activity, while KDM6A, KDM7A, KDM3B, and KDM5C were linked to histone demethylase activity (Figure 2F). These genes were found to be highly expressed in the peripheral blood of the AQP4-ON group compared with the other two groups (Figure 2H). Interestingly, these genes also occupied central positions within the protein-protein interaction network of key genes (Figure 2G).

### Clinical relevance of key genes

**FIGURE 3:**
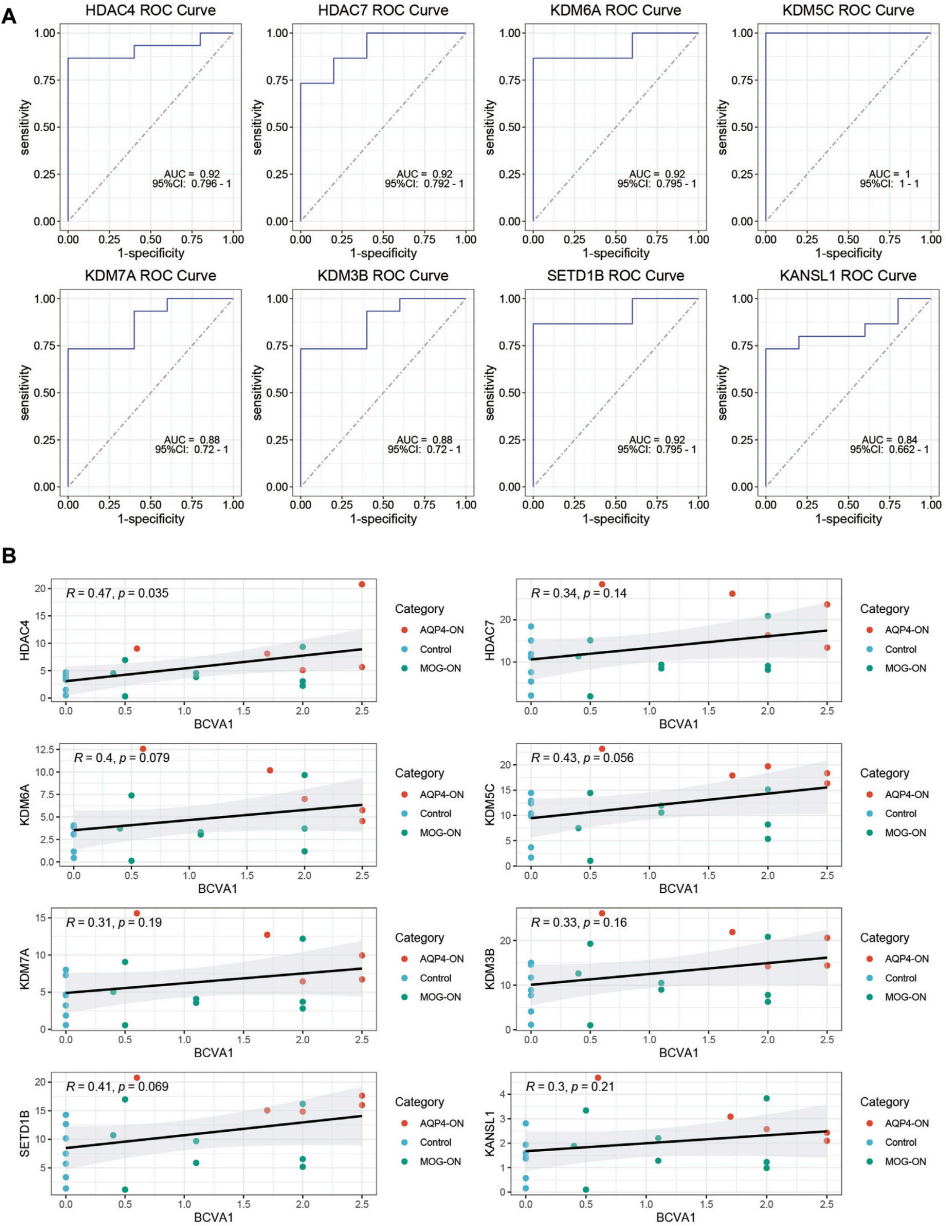
ROC Curve Analysis of Key Genes. **(A)** ROC curves for the eight investigated genes, with AUC values indicating their diagnostic potential as biomarkers for AQP4-ON. The 95%CI of AUC values were also marked in the ROC plot. **(B)** Clinical relevance of the eight investigated genes. The correlation between TPM levels of the genes investigated and initial-onset log MAR of the patients were assessed using Spearman correlation analysis, and P values were calculated accordingly.

To evaluate the diagnostic potential of the investigated genes as biomarkers for AQP4-ON, we conducted a ROC curve analysis. The findings indicated that all eight genes studied demonstrated the capacity to serve as biomarkers for distinguishing AQP4-ON. Notably, HDAC4, HDAC7, KDM6A, and KDM5C exhibited relatively high AUC values (0.92, 0.92, 0.92, and 1.00, respectively), suggesting that these genes, which are implicated in histone modification, are highly accurate in differentiating patients with AQP4-ON from those with MOG-ON and healthy controls (Figure 3A). Moreover, positive correlations between the expression level of key genes and the severity of the optic neuritis at its initial presentation could be found (Figure 3B).

## Discussion

With the progress of molecular biology and bioinformatics, current understandings of AQP4-related and MOG-related optic neuritis have transcended the realm of autoantibody discovery, advancing into the molecular mechanisms and the realm of epigenetics (Carnero Contentti and Correale, 2021; Mehmood et al., 2023). Peripheral blood transcriptomics, favored for its accessibility and reflectiveness of the patient’s immunological molecular biology, has become a milestone in this research domain (Chen et al., 2023; Wylezinski et al., 2024). In this study, we applied Weighted Gene Co-expression Network Analysis (WGCNA) to an in-depth transcriptomic analysis of peripheral blood samples from patients with AQP4 antibody and MOG antibody-positive optic neuritis. We aimed to identify key genes and biomarkers associated with the pathogenesis of AQP4 antibody-positive optic neuritis, explore the molecular expression differences and pathogenic mechanisms between AQP4-positive optic neuritis and MOG antibody-positive optic neuritis, and seek transcriptomic markers with a degree of specificity.

The WGCNA analysis unveiled several gene modules significantly correlated with the clinical phenotypes of AQP4 positive optic neuritis. Particularly, the brown module (MEbrown) demonstrated a close association with the phenotype of AQP4-positive optic neuritis. The positive correlation observed between this module and the extent of visual acuity decline further underscores the potential role of these genes in NMOSD. We thus filtered genes that occupy central positions within the network and are closely related to the disease type, in an attempt to uncover unique key molecules and biological processes involved in the pathogenesis of AQP4-positive optic neuritis.

Functional annotation analysis of the identified key genes revealed their involvement in biological processes such as histone modification, microtubule polymerization/depolymerization, and transcriptional corepressor activity, all of which played crucial roles in immune cell activation, migration, and neuroinflammatory responses. The roles of genes like HDAC4, HDAC7, and SETD5 in histone deacetylation and transcriptional regulation, alongside the functions of KDM6A, KDM7A, KDM3B, and KDM5C in histone demethylation, suggest that epigenetic alterations, particularly histone demethylation, and deacetylation-mediated transcriptional activation, may play a key role in the immunoregulation and neurologic damage in AQP4-ON and NMOSD.

Our findings are consistent with current research on the molecular mechanisms underlying AQP4-ON and NMOSD, highlighting the roles of epigenetic changes and cellular immunity in the disease (Guo et al., 2023; Mehmood et al., 2023). Notably, the majority of key genes identified in this study are enriched in epigenetic modifications such as histone demethylation and acetylation, a significant discovery suggesting a crucial role for epigenetic changes in AQP4-ON. Moreover, It has been suggested that histone modification acted as the upstream molecular event preceding the activation of multiple pro-inflammatory pathways, for example, the NF-κB pathway(Piatek et al., 2022; Piatek et al.,2024; Guo et al., 2023), and changes in the histone-modifying activities may have a potential implication on the disease (Zhang et al., 2010). The potential roles of these genes in mediating epigenetic alterations in the immunoregulation and neurologic damage of AQP4-ON warrant further experimental validation.

Despite the novel insights provided by our study, several limitations must be acknowledged to ensure a comprehensive interpretation of the results. The modest sample size, while sufficient for the preliminary analysis presented, may restrict the generalizability of our findings. Future studies with larger and more diverse cohorts will be essential to validate the diagnostic and prognostic significance of the identified biomarkers. Additionally, our analysis, primarily based on peripheral blood samples, may not fully encapsulate the central nervous system-specific changes in NMOSD. Incorporating analyses of cerebral spinal fluid samples could offer a more nuanced understanding of the disease’s molecular pathology (Qin et al., 2024). Moreover, the observational nature of our study necessitates further experimental research to elucidate the biological functions and mechanisms of action of the identified key genes. Clinical cohorts and animal models will be invaluable in this regard, providing a platform to explore the specific roles of these genes in the pathogenesis of NMOSD. As we look into the future, the potential of these genes as therapeutic targets presents an exciting avenue for investigation, one that could yield transformative approaches to the treatment of NMOSD.

In conclusion, our study represents a significant step forward in the molecular characterization of different types of optic neuritis, offering a foundation upon which future research can be built. The identification of potential biomarkers and the exploration of their roles in disease pathology lay the groundwork for a more personalized and targeted approach to the diagnosis and treatment of NMOSD.

## Data Availability

The data presented in this study are deposited in the GEO depository (www.ncbi.nlm.nih/geo), accession number GSE226808.

## Supplementary material

SUPPLEMENTARY TABLE S1 Clinical Phenotype data of participants in this study.

SUPPLEMENTARY FIGURE S1 Sequencing data before **(A)** and after **(B)** z-score normalization. The data were presented as log2(TPM+1).
