# Supplementary material for: Brief research report: WGCNA-driven identification of histone modification genes as potential biomarkers in AQP4-Associated optic neuritis
Source: Front Genet. 2024 Aug 22;15:1423584. doi: 10.3389/fgene.2024.1423584 (PMC11374599; doi:10.3389/fgene.2024.1423584)
Supplement: Supplementary file 1 [file Table1.DOCX]

|  | Type | BCVA1  (logMAR) | BCVA2  (logMAR) |
| --- | --- | --- | --- |
| H1 | Healthy Control | **0** | 0 |
| H2 | Healthy Control | **0** | 0 |
| H3 | Healthy Control | **0** | 0 |
| H4 | Healthy Control | **0** | 0 |
| H5 | Healthy Control | **0** | 0 |
| H6 | Healthy Control | **0** | 0 |
| H7 | Healthy Control | **0** | 0 |
| A1 | AQP4-ON | **3.5** | 3.5 |
| A2 | AQP4-ON | **1.7** | 2 |
| A3 | AQP4-ON | **2** | 0.4 |
| A4 | AQP4-ON | **0.6** | 0 |
| A5 | AQP4-ON | **2.5** | 0.2 |
| A6 | AQP4-ON | **2.5** | 0.4 |
| M1 | MOG-ON | **0.4** | 0.2 |
| M2 | MOG-ON | **0.5** | 0.1 |
| M3 | MOG-ON | **1.1** | 0.4 |
| M4 | MOG-ON | **1.1** | 0.2 |
| M5 | MOG-ON | **2** | 0 |
| M6 | MOG-ON | **2** | 0.1 |
| M7 | MOG-ON | **2** | 0.9 |
| M8 | MOG-ON | **0.5** | 0.2 |

Table S1
