# Supplementary figures and images for: Brief research report: WGCNA-driven identification of histone modification genes as potential biomarkers in AQP4-Associated optic neuritis
Source: Front Genet. 2024 Aug 22;15:1423584. doi: 10.3389/fgene.2024.1423584 (PMC11374599; doi:10.3389/fgene.2024.1423584)

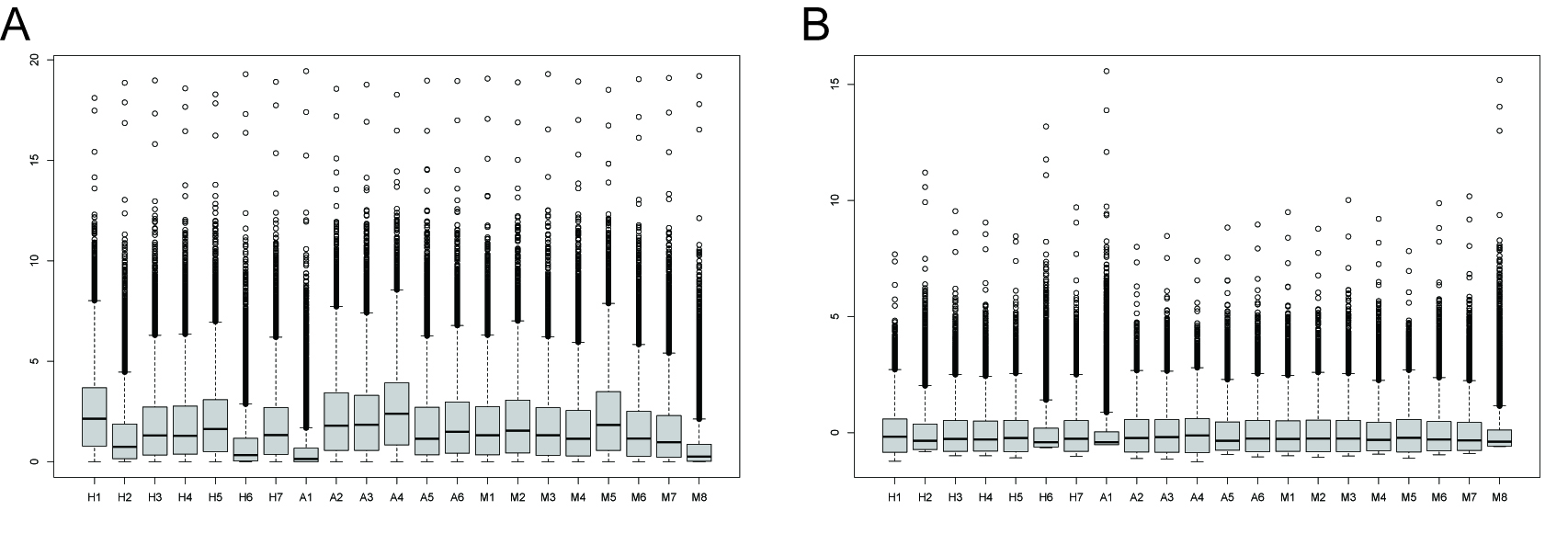

Supplement: Supplementary file 2 [file Image1.JPEG]
